# Machine learning based customer churn prediction in home appliance rental business

**DOI:** 10.1186/s40537-023-00721-8

**Published:** 2023-04-05

**Authors:** Youngjung Suh

**Affiliations:** LG Electronics Inc, Yeongdeungpo-Gu, Seoul, 07336 South Korea

**Keywords:** Big data applications, Customer churn prediction, Machine learning, Churn in rental business, Feature selection, Customer retention management

## Abstract

Customer churn is a major issue for large enterprises. In particular, in the rental business sector, companies are looking for ways to retain their customers because they are their main source of revenue. The main contribution of our work is to analyze the customer behavior information of actual water purifier rental company, where customer churn occurs very frequently, and to develop and verify the churn prediction model. A machine learning algorithm was applied to a large-capacity operating dataset of rental care service in an electronics company in Korea, to learn meaningful features. To measure the performance of the model, the F-measure and area under curve (AUC) were adopted whereby an F1 value of 93% and an AUC of 88% were achieved. The dataset containing approximately 84,000 customers was used for training and testing. Another contribution was to evaluate the inference performance of the predictive model using the contract status of about 250,000 customer data currently in operation, confirming a hit rate of about 80%. Finally, this study identified and calculated the influence of key variables on individual customer churn to enable a business person (rental care customer management staff) to carry out customer-tailored marketing to address the cause of the churn.

## Introduction

Customer churn refers to customers terminating their relationship with a company that provides products or services. Churn prediction refers to detecting which customers are likely to leave or cancel a subscription to a service. Churn prediction has become one of the most important marketing campaigns nowadays as the main strategy to survive in the fiercely competitive market of major companies in developed countries.

To enhance the competitiveness of companies, three main strategies have been proposed [1]: (1) acquiring new customers, (2) increasing sales to existing customers, and (3) extending the customer retention period. However, by comparing the importance of these three strategies based on the value of return on investment (ROI) for each, it was found that the third strategy was the most profitable [1–3]. To enhance corporate competitiveness through the third strategy, customer churn should first be predicted to reduce the possibility of churn, to bring economic benefits to the enterprise [7]. Other related studies have emphasized the need for strategies to maintain customers arguing that customer maintenance costs are lower than the cost of attracting new customers [4, 5, 8, 9].

In the past, companies tried to understand the reasons behind churn numbers and tackled these factors with reactive action plans. To assess in advance whether a specific customer is likely to leave the company, companies can strategically employ machine-learning-based customer churn prediction models to take proper action in time to prevent customer churn. Research on customer churn prediction has been mainly conducted on telecommunication companies and financial domains over the past few decades [14–24].

Meanwhile, recently, due to the prolonged COVID-19, non-face-to-face customer management and marketing have become increasingly important across all industries. As the time spent indoors increases, interest in and requirements for subscription services for home appliances have increased. In this regard, it is important to manage a variety of non-face-to-face visiting services and conduct marketing based on customer characteristics. Accordingly, predicting the possibility of customer churn has become one of the main survival strategies in the home appliance rental business. Thus, there is room for opportunities for data-based customer churn prediction modeling research in the home appliance rental business.

A wide range of subscription data collected by companies can contribute significantly to the transition from a follow-up to a proactive strategy. The advent of machine learning technology has helped companies use this rich data to resolve customer churn in a much more effective and systematic manner. However, studies on establishing strategies through analysis of actual contract data and service use of home appliance subscribers, have been insufficient. Few studies have focused on quantifying churn risk related information by analyzing the characteristics of home appliance subscribers.

Therefore, this study examines churn prediction models for proactive churn management in home appliance rental businesses. The purpose of this study is to determine which service subscribers to the rental care solution business are likely to churn, what the reasons are for the churn, and thus, to determine whom and what retention strategy to target. To this end, an integrated analysis was performed on customer contract information, demographic information, transactions, and customer-firm interaction data on rental service subscriptions. Machine-learning techniques were applied to churn prediction problems using the analysis results.

In the rental service business of the electronics company, which inspired this study, post- and passive defense activities have encouraged customers to request termination of the agreement instead of waiting until maturity. However, successful defense coverage has not been as high as expected. In addition, the level of defense activities has not been differentiated at the customer level but has been limited to cluster-level (e.g, defense strategies in large categories such as months of use, product lines, etc.). Therefore, the goal of this study is to propose a machine-learning-based customer churn defense tool that effectively learns and predicts signals pertaining to the possibility of customer churn, enabling active churn management.

The contributions of this paper are in the following three aspects.

1. Feature engineering considering domain knowledge: major valuable churn predictors were identified for customer retention by considering domain knowledge, such as customer subscription history information, ranging from contract application to installation, operation, and change (termination and renewal). In other words, in data processing, rather than grasping the meaning of the attribute from the column description or performing preprocessing using machine learning, the feature was derived based on a discussion and consultation with the rental care staff while developing the prediction model.
2. Obtaining predictive performance using the actual operating data-based learning model: In this study, the churn prediction model was verified on an actual operational dataset, not a benchmark dataset [28], to represent actual customer service usage and needs. Modeling was conducted to predict the risk of churn for each customer based on machine learning using a real-world dataset (84,000 accounts of water purifiers). The performance of the churn prediction model was evaluated as about 90% using the F-measure and AUC score. In addition, the inference performance of the prediction model was verified by monitoring the actual contract status change of the account to be tested (250,000 accounts), demonstrating about 80% hit rate as a result of 4-month monitoring
3. Analysis of the major features of individual observations contributing to an explanation of the predictive model: Customer-specific churn factors for an individual customer were analyzed to enable detailed target care (defense) compared to the existing group-level defense strategy. It is expected that more efficient manpower operation and cost optimization will be possible through target marketing by utilizing customer churn risk scores as well as information on churn factors. The results of this study show that rental care service companies can introduce customized contract extension activities depending on the risk of churn for each customer. That is, rather than relying on call agents to identify and defend against customer churn over the phone, machine learning can be used to predict high-risk customers.

The remainder of this paper is organized as follows. In "Related work" Section. the related studies on customer churn prediction are reviewed. In ‘‘Study design’’ Section study design for modeling is introduced. In "Feature analysis and selection" Section, the details of the modeling for customer churn prediction are presented. In "Evaluation results" Section the experimental setting is described and an analysis of the experimental results is presented. The final section concludes the study and offers further research directions.

## Related work

Predicting customer churn is important for customer retention, and essential in preventing huge losses in many industries. Currently, as the need to predict and prevent customer churn in various domains is increasing, many data-mining and machine-learning technologies are being used for this purpose [41]. In addition to building a stable model that can predict customer churn, it is also very important for companies to efficiently retain customers to avoid heavy losses [10–13].

### Technical methods for churn prediction

First, regarding the methodology in the field of marketing, which most directly deals with customer retention as an important issue, there are many studies ranging from simple recency, frequency, and monetary (RFM) models [25] to ensemble machine learning methods [15, 26] such as random forests, which focus on finding the best way to predict customer churn. Machine learning methods outperform traditional statistical methods in predicting churn [27]. Buckinx and Van den Poel [35] proposed a churn definition for the non-contractual setting, to predict who is likely to churn based on the RFM model that they designed using transactional data and demographic information. They evaluated the model using logistic regression, neural networks, and random forests obtaining an AUC score of 83%. Migueis et al. [36] developed an RFM-based predictive model using customer purchase histories and evaluated it using machine learning techniques such as logistic regression. The performance measures of their prediction model were presented in terms of AUC for the top 1st, 5th, and 10th percentiles.

Technical challenges are mainly prediction performance of classification algorithms and class imbalance problem. Previously, researchers employed single classification methods for customer churn prediction. Ensemble-based classification algorithms have been recently developed [50–52]. Recently, Liu R et al.[47] proposed an ensemble learning technique fully incorporating clustering and classification algorithm for customer churn prediction. Another issue is class imbalance in customer churn prediction. For this, previous studies have mainly applied Synthetic Minority Over-sampling Technique (SMOTE) [48, 49]. Recently, hybrid resampling methods have been proposed as a more effective method to tackle imbalanced data. Kimera T [46] proposed hybrid resampling such as SMOTE-ENN and SMOTE Tomek-Links as novel and effective resampling methods for customer churn prediction.

In this non-contractual setting, where it is necessary to define who is likely to churn, churn prediction studies show excellent prediction performance by taking advantage of deep learning methods as well as traditional machine learning algorithms. Seymen et. al. [30] applied a deep learning algorithm to a dataset of supermarket transactions in the retail domain to predict customer churn and compared the performance to other well-known churn modeling approaches. Dingli et. al. [37] compared two deep learning algorithms, the convolutional neural network and the restricted Boltzmann machine, to predict churn in the retail sector. Among these studies, some perform churn prediction modeling using deep learning methods although not in the non-contract setting. Alboukaey et al. [38] proposed a daily churn prediction method, modeling daily behavior as multivariate time for churn prediction. A statistical model, the RFM model, long short-term memory (LSTM) model, and convolutional neural network (CNN) model were also applied to a mobile communication dataset.

The rental care service addressed in this study is a customer churn prediction problem in the contractual setting sector. The features related to customer contracts and service usage history used for the modeling are derived from data which can be correlated and thus do not display the continuity that many deep learning methods generally assume. Compared to artificial neural networks that successfully classify problems related to nonlinear decision-making hyper-surfaces but are difficult to interpret, tree-based machine learning, predicts customer churn in an intuitive and easy-to-interpret way based on customer rental service usage history information. For example, if there is a lot of repair history caused by defective products, there is a high probability of customer churn.

In other words, when performing churn defense marketing using the customer churn prediction model, the performance of the predictive model is also important; however, it is more important to discover important attributes that marketers can intuitively understand, utilize, and interpret. Therefore, in this study, a method was adopted to determine important attributes through feature engineering, whereby a machine-learning algorithm was applied to the discovered features. In machine learing models, feature selection plays a significant role to improve the classification accuracy. Lalwani et al. [44] applied gravitational search algorithm(GSA) to perform feature selection and to reduce the dimension of data-set for better understanding of the data. They also both optimized algorithms and achieved better results by using the power of ensemble learning. In our subsequent study, the customers' water purifier usage behavior data, such as daily usage frequency, usage time, and amount of water are to be added as features to predictive modeling, to further improve the predictive power of customer churn. It is worthwhile to consider the feature engineering method like GSA in subsequent studies because the data sources are huge in size increasing complexity in dealing with them. Another study [45] used customer social network in the prediction model by extracting Social Network Analysis (SNA) features such as degree centrality measures, similarity values, and customer’s network connectivity for each customer. They achieved good improvement in AUC results by using SNA features. Customer information like Social Network from an external data source is valuable in giving more different perspectives about the customers. We are in an alliance process to analyze and utilize the integrating information by combining the pseudonymous information of the external company and the company, and the predictive performance is expected to improve through our future combined analysis.

A recent study [34] proposed a customer churn prediction model based on deep learning and included an analysis on the cause of predictive power using an autoencoder and an unsupervised learning model. Another study [21] mentioned the importance of feature extraction in the churn prediction model but suggested that the deep learning method without selecting or extracting features was as successful as the traditional method. Therefore, it is worthwhile to consider deep learning in subsequent studies.

### Application domain for churn prediction

Customer churn is mainly relevant to contract setting sectors such as telecommunications, banking, and insurance, where customers must enter a contract to receive services from the company. Therefore, in churn modeling in this setting, the customer who cancels the contract is classified as a churner and the customer who continues to receive the service is a non-churner. However, in the case of non-contract settings, such as retail and games, before proceeding with churn modeling, the definition of churn should be clarified. The literature review of this study focuses on research on churn in the contract setting sectors.

In the telecom domain, the mobile communication market is slowing down because of market saturation, and the launch of the latest smartphones causes customers to opt out of their contract. To address the issue of customer churn in such a competitive market, it is vital to first identify the key factors that affect customers’ decisions by gathering reliable customer information and establishing a more accurate customer churn prediction model. Two recent studies address this issue [14, 31]. In recent years, customer churn management has emerged as an important task in customer relationship management (CRM) in the mobile communication industry [6]. Under these circumstances, mobile communication companies are making great efforts to maintain customers by modeling patterns of customer behavior by applying data-mining techniques to existing customer data to establish effective marketing strategies. Numerous studies have recently been conducted on the issue of churn rates and marketing costs. To increase business profits by more than several times, customers who can leave are classified in advance and intensive marketing activities are conducted to retain them [16–18].

In the financial sector, future behavior is predicted through customers’ past financial transactions, and the results are used to develop various customer management tasks and new products. Looking at previous studies of customer churn prediction models in the financial sector, research on prediction models was conducted in Korea, mainly in banks and the credit card industry with a large number of customers because academic access to financial data is relatively difficult compared to overseas [20]. Most of the preceding studies were divided into those that present a method of improving predictive performance by applying various machine learning algorithms [21, 43] and those that segment the customer marketing indicators using predicted results [22–24].

Meanwhile, the traditional business model of water purifier manufacturing and sales was introduced in Korea in the late 1980s because of an urgent demand for better quality drinking water. However, from the beginning traditional model was not successful because not only the initial purchase cost was high, but also the demand for frequent maintenance required for optimal operation was relatively high. Thus, the water purifier manufacturer made all efforts, such as regular maintenance and repair, to switch to a rental business model to prevent failures or problems in products rented during the contract period [29]. As mentioned earlier in the studies [14, 15] related to the mobile communication domain, customer retention costs were less than the cost of acquiring new customers; therefore, the strategy for retention adopted by companies was considered equally important in the home appliance rental business. At the beginning of a business, it is necessary to focus on attracting new customers. As the number of customers who have passed the mandatory use period increases, the risk of churn increases; therefore, intensive customer management is required. Research has been conducted on the potential of the rental business model to enhance competitiveness and sustainability [29], but few researchers have concentrated on quantifying customer churn risk by analyzing the characteristics of rental service subscribers.

To address these problems, in this study, the main features of customer maintenance (customer churn defense) in the rental business were identified starting with the contract application stage all the way to the installation, operation, and change (termination and renewal) of the customer’s subscription history information. Machine learning-based modeling was performed to predict customer-specific churn risk based on the identified features, and model explanation-based churn factor information was derived to differentiate target care and marketing for each customer with churn in mind.

Specifically, two questions were of interest in customer churn prediction. First, the attributes related to customer churn, the rental contract details, service usage history, and history of contact with customers related to churn were determined. Second, automatically identifying the customers considering leaving was also of interest. As usage problems may gradually change over time with repeated usage behaviors having negative consequences, it is important to identify the signals for churn as early as possible so that appropriate interventions can be introduced. Thus, the following two questions arise,

• Can we identify features that correlate well with customer churn?

• If so, what combination of features/models has the best prediction accuracy? How successful is the predictive model with the selected features in identifying, with a good hit rate the customers with a high risk for churn from among the actual operating customer data?

## Study design

Before proceeding with the customer churn prediction modeling work, the following two questions must be answered.

- First, how can a churner be defined as discriminating from others in terms of contract details?
- Second, what analysis scenarios (hypotheses) for feature selection indicate the characteristics of churning customers?

### Classification of customers and analysis scenarios

Rental care service customers, during the process from contract start to expiration and extension, can have the status of “in contract,” “terminated,” and “expired.” The contract types consist of “newly contracted” and “re-contracted.” The three customer groups classified by contract type and contract status are shown in Table 1.

**Table 1:** customer groups classified by contract type and contract status

Customer Groups	Description
Churn	Customers who left the service after contract termination
Re-subscription (mid-term renewal and expiration renewal)	• Customers who re-subscribed after termination In case of renewal in the middle of the contract (creating a new contract with a new device): it is often recommended to sell a new model or replace it with a model with different specifications while renewing the contract • In case of renewal at the time of expiration: the contract is renewed due to the expiration of the contract period
Contract maintenance	Customers who continued to use the service (customers who maintain contracts with existing devices)

To perform machine learning based on supervised learning, labeled data with correct answers are required. The customer group classified as “cancellation (churn = Y)” included customers who canceled mid-term and customers who did not renew their contract after the contract expired. In addition, the customers whose current contract status is active were not labeled as “renewal (churn = N)” because they can cancel any time until the expiration date. Therefore, the right answer dataset was created by assigning the label “renewal (churn = N)” to customers who renewed the contract after termination in the middle of the contract or customers who renewed the contract after termination at the time of expiration.

In the case of re-entry customers in the middle of the contract (contract extension), there are cases where customers voluntarily want to use the new product model when renewing. In most cases though, contracts are extended through a discount policy. However, there was no way to differentiate customers who renewed with a discount policy from those who did not based on the available data. Therefore, it is necessary to consider whether to classify a mid-term renewing customer as a renewing customer. Although they were included as recontract customers in this study, it would be meaningful to categorize them differently in future studies. Then an analysis scenario was established with a businessperson (rental care customer management staff) to select attributes that could affect customer churn, as shown in Table 2.

**Table 2:** Questions for analysis scenario

Questions	Analysis items
Which customers mainly churn?	• Customer demographics (gender, age, region, etc.) • Rental contract details and service usage history Contract application and installation stage: order information (model, quantity, order type, sales channel, etc.), contractor/acquirer, installation location, etc Contract operation stage: rental fee billing/collection information, payment method, discount program, commitments (dates and amounts), care service (visit date and time, visit cycle, service content, and quality of the manager), etc
When does churn usually occur?	• Analysis of customer churn rate by contract time and number of months of use
Is there any correlation between contact history and churn?	• Analysis of correlation between the number of calls and the number of customer churns • Analysis of correlation between the number of detailed call types and the number of customer churns

### Customer selection and Y/N labeling

**Fig. 1:**
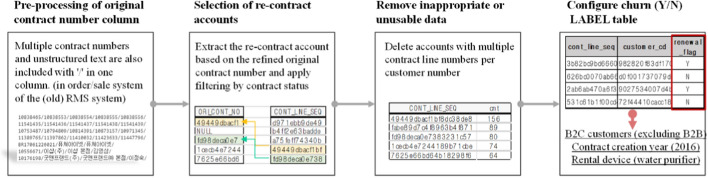
Detailed logic of creating customer churn (Y/N) LABEL table

Here, the process of selecting target customers for training the classification model is described along with the labeling process of Yes and No to construct a set of correct answers for supervised learning. Among the accounts contracted in 2016, approximately 84,000 customers who rented water purifiers were selected for the analysis. The selection criteria were as follows. Contracts that were operated until maturity and those that were affected at a minimum by the company’s intentional re-rental policy were selected. To create the Y/N LABEL table mentioned in the previous section, target customers were classified as “Customer group who left after canceling the service (churn = Y)” and “Group that re-entered after a cancellation (churn = N).” The details of the logic for classifying into two groups is shown in Fig. 1 and outlined as follows:

First, “renewing customer master table” was created by only extracting the renewing customer account which had the original contract line number. Among all the canceled accounts, the re-contract account was extracted by selecting the contract line number that matches the original contract line number in the “renewing customer master table.” Next, a filtering condition was applied to the analysis target range. The condition is to filter the renewal contract account creation date based on termination and expiration dates. To this end, using the domain knowledge of the businessperson, the criterion for “customer churn” was defined as the case where the contract is not renewed within “one year after expiration” or “6 months after termination.” In addition, customer data with multiple contract line numbers per customer were deleted. This is because outlier data such as customer accounts remain in the initial system. Finally, only B2C customers who created contracts for water purifiers in 2016 were selected. Thus, a customer churn LABEL table with a pair of contract line numbers and churn (Y/N) was constructed.

In general, the number of retained customers is much larger than the number of churning customers. For example, it is assumed the data consists of 90% retained customers and 10% churn customers. If the predictive model is trained with the data tagged with the Y label of the retained customer, the model outputs have an accuracy of 90%. Although the model is said to be highly accurate in this case, it may not be effective in identifying the characteristics of churning customers. Therefore, the ideal method is to adjust the churned and retained data in a similar proportion to increase the likelihood of obtaining a decision boundary for a minority group [40].

Our LABEL dataset contained a total of 84,000 customers of which 23,000 (27%) were renewing customers and 61,000 (73%) were churns. The target variable for this assessment is the “churn” feature. Because of the characteristics of the water purifier rental service, which has a relatively high percentage of churning customers compared to other domain services, the labeled data have a sufficient Y-label data ratio to identify the characteristics of churning customers. According to the knowledge of the businessperson, a certain percentage of customers renew the contract on the expiration date. These percentages come from the rental fee discount policy applied to those who have renewed contracts within one year after expiration. Therefore, when calculating based on the complete data of the contract period over five years, even if there is a difference by year, on average, the ratio of customers who cancel and renew contracts is said to be about 50:50. The proportion of canceling customers was about 70% at the time of generating the learning data accounts, excluding those renewing with the discount policy.

## Feature analysis and selection

In this section, the process of analysis and feature selection influencing the customer’s decision to leave are introduced.

### Data preparation(pre-processing)

The domain knowledge and intuition of the rental care service staff are essential in performing the feature engineering process to extract the optimal feature variables to be used in predictive modeling. Therefore, the user interface screen of the rental care customer management system currently in operation was investigated and the screens containing the data needed for the analysis were selected together with the staff. Approximately 1400 customer-related attributes were selected from each screen. Next, approximately 340 attributes essential for hypothesis verification of the previously established analysis scenarios (in ‘‘Study Design’’ Sect) were selected.

Then, an analysis data mart for the learning model was constructed by combining the analysis variables selected above (29 tables, 340 attributes) with the Y/N LABEL from the correct answer set data. The analysis tables were divided into two types: a snapshot table group that matches 1:1 by contract line number, and a history table group that exists as 1: N by contract line number. First, in the case of a non-history table, an analysis table was created by joining the necessary attributes for each of the above 29 tables based on the two primary attributes (CONT_LINE_SEQ and Churn Y/N) of the Y/N label table. For the history table, an analysis table was created by creating a derived variable to which the summary logic of the corresponding attribute was applied. An example of such a history table is one that stores the date and time of a customer’s visiting service over a specific period.

**Fig. 2:**
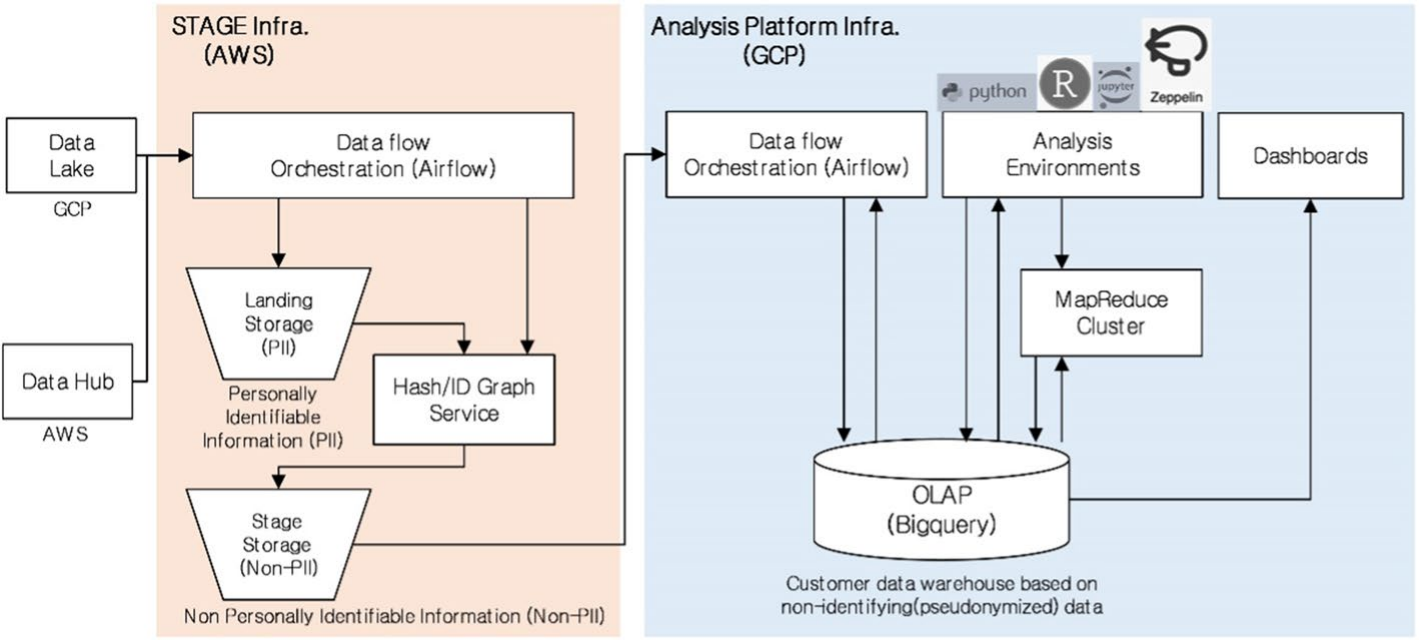
Big data platform for customer data analysis

Datasets in the analysis tables may contain values that are often missing due to data corruption or failure to record. With the help of the domain knowledge of the rental care service staff, other attributes that could be used in place of the attributes deleted due to missing values were also selected. Figure 2 shows the infrastructure configuration that collects and pseudonymizes all identification data in AWS and transmits all pseudonymized data to a GCP-based customer data analysis platform. Because customer identification information was pseudonymized due to privacy issues in the data analysis platform shown in Fig. 2, attributes such as customer age could not be used. In addition, attributes such as gender and address were not available because of the regulations that required that customers’ personal information to be discarded. Through the univariate descriptive statistical analysis, the attributes excluded due to the high NULL ratio were as follows: customer address, account suspension date, housing type code, whether to combine products, etc. In summary, approximately 200 attributes among 340 were selected based on the availability of each attribute through descriptive statistics and the knowledge of the staff.

### Feature selection

For feature selection, feature engineering techniques and the analysis results are described. Firstly, Principal component analysis (PCA) was used as a feature selection technique. The basic idea when using PCA as a tool for feature selection is to select variables according to the magnitude (from largest to smallest in absolute values) of their coefficients. But the problem with using PCA is that only linear relationships are considered. So, a complementary method for feature selection is required. With EDA, we aimed to find the variables that may have been missing from PCA but are statistically essential.

#### Analysis methods

**Fig. 3:**
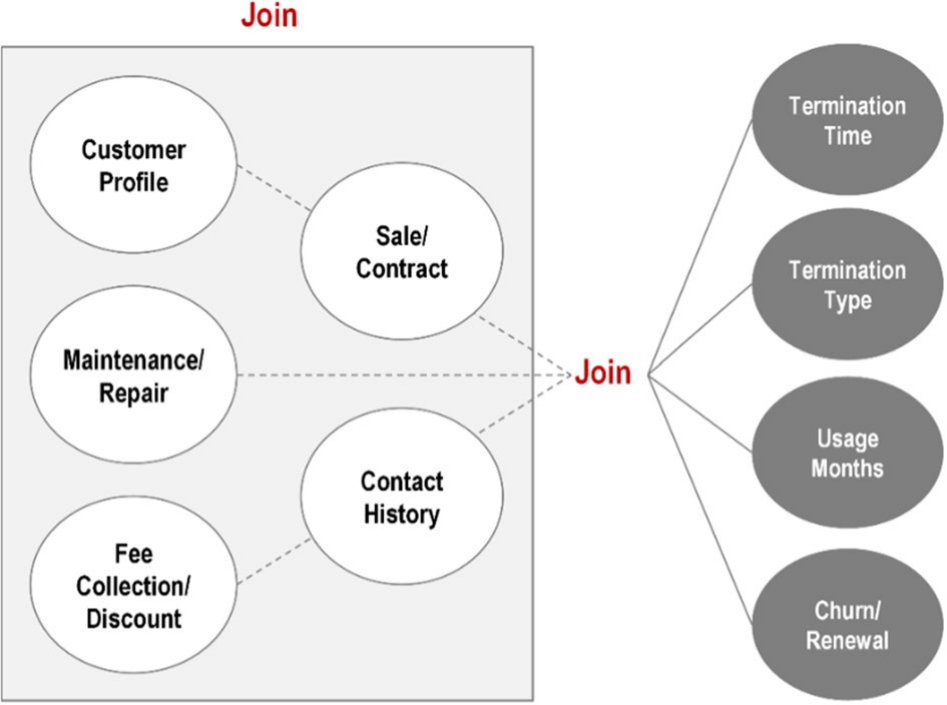
Combination analysis items for each major characteristic information of a customer to create a derived variable

The main attributes of the customer in the first feature set included information on sales/contract, payment, installation, contact history, maintenance (visit history), commitment, and discount. First, the key attributes were selected by checking whether they showed a statistically significant difference with respect to the Y/N label. To create meaningful derived variables, the attributes were analyzed by combining them with termination time and termination type, as shown in Fig. 3. Therefore, among the features created based on 200 attributes, 130 major features were selected with statistically significant differences in distinguishing between the canceled and re-contracted customer groups. The following attributes were excluded: prepayment application, purchase path, subscription path, installation cost, installation point name, sales type, contractor change, etc.

The approximate performance of our machine-learning algorithm was checked using approximately 130 feature sets. The prediction model performed better than expected because of the features created from the results of the customer’s action on termination, such as reason for application for cancellation, date of receipt of cancellation. A feature corresponding to a post-signal was excluded from the feature set because it is not suitable for a predictive model that detects customer cancellation signs in advance.

In addition, some of the 130 features were regenerated through the following variable reprocessing process to increase the discriminating power. First, considering the category ratio of the categorical variable values, only the top N% or higher values were created as feature variables. Next, a feature with a hierarchical structure between attributes was created as a new type of variable based on hierarchical information, and among them, only the variables with a significant difference between Y/N labels were selected. In addition, summary variables were regenerated by changing the aggregation period to the most recent year (a period that increased the significant difference in Y/N labels) rather than the entire period.

**Fig. 4:**
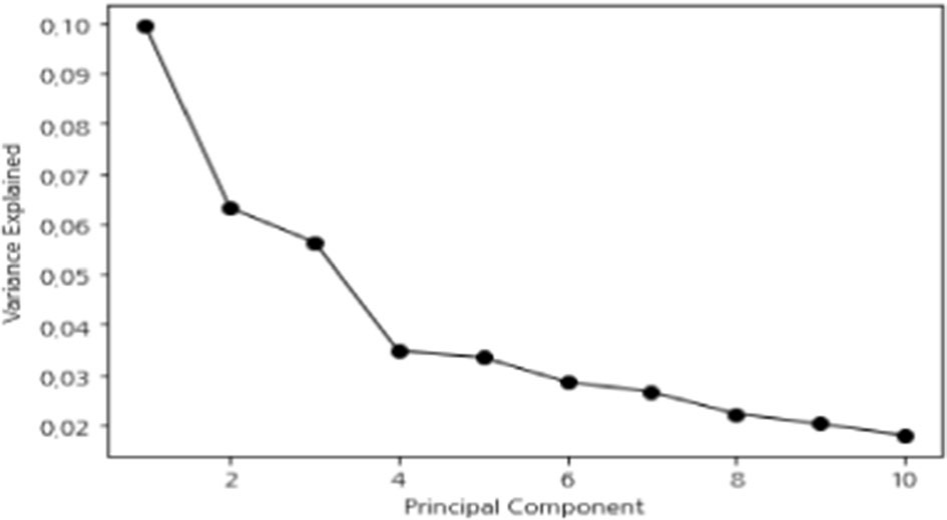
Variance explained of principal components in PCA

Principal component analysis (PCA) was used as a feature selection technique. First, features exceeding 10% for the top four principal components were investigated. As shown in Fig. 4, it was confirmed that the first PCA described a variance of 0.993, and the second and third components accounted for a variance of 0.063 and 0.056, respectively.

**Fig. 5:**
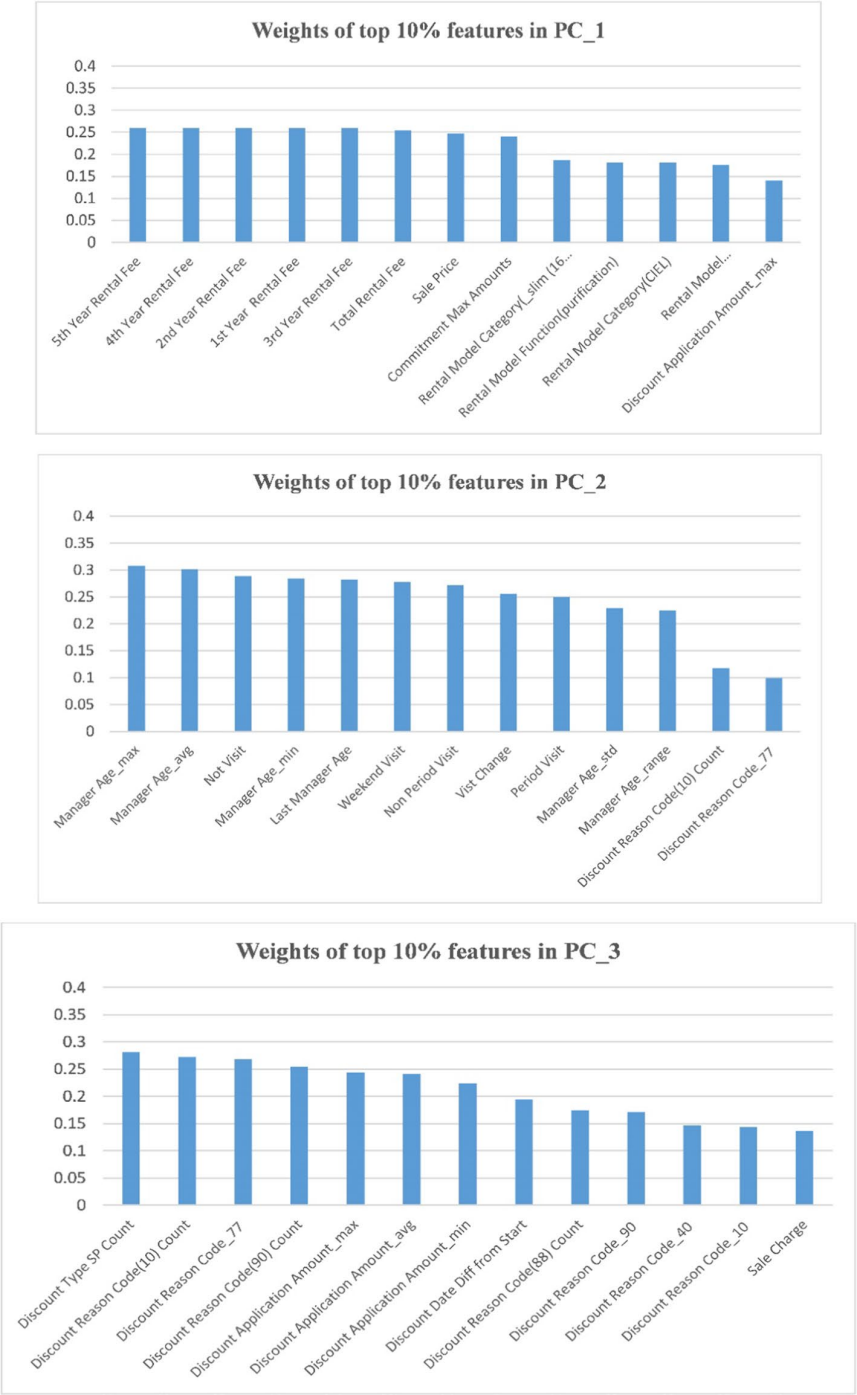

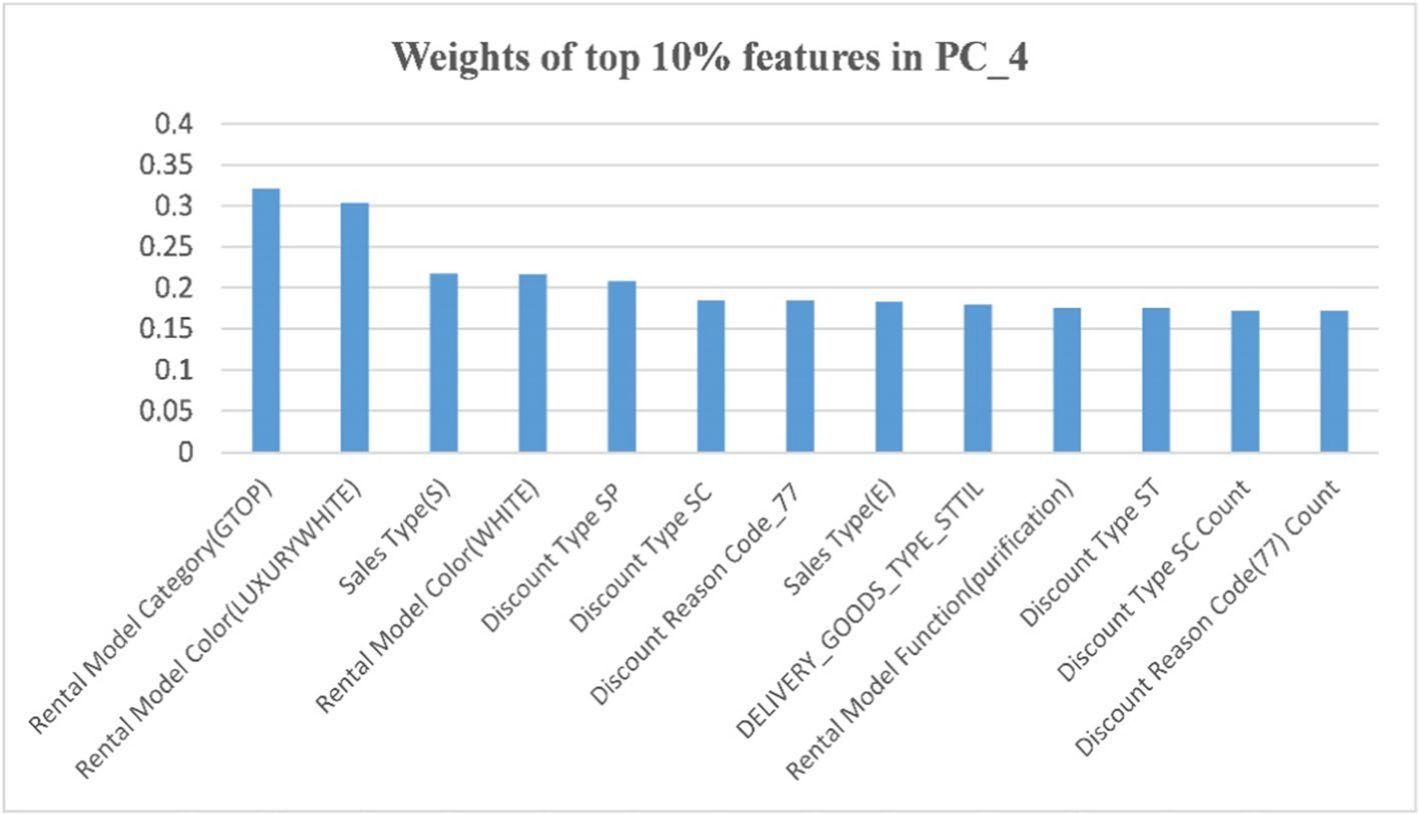
Weights of the top 10% of features in the top four principal components (**a**) weights of PC_1 (**b**) weights of PC_2 (**c**) weights of PC_3 (**d**) weights of PC_4

In addition, as shown in Fig. 5, several main themes appearing in the features related to individual principal components were identified, namely, rental fees, commitment amounts, model category/function/color, manager age, visit history, discount reasons/type/amount/date, and sales type.

Features for customer contact history, which are considered as important attributes for hypothesis analysis were found, from features exceeding 40% among the top four principal components. PCA constructs each principal component as a linear combination of the original variable by recognizing the importance of the feature from the corresponding weighting coefficient of the original variable. Therefore, the final feature candidates commonly found in both features exceeding 40% for the top four principal components of PCA and those showing significant differences between Y/N groups in exploratory data analysis (EDA) were selected. It left us with 80 of the 130 features.

#### Exploratory data analysis results

This section introduces the analysis results, focusing on the significant ones among the EDA results of the 80 features selected above. In the results below, “N” of the renewal_flag means “churn” and “Y” means “renewal,” respectively for customers who leave and renew their contract.

**Fig. 6:**
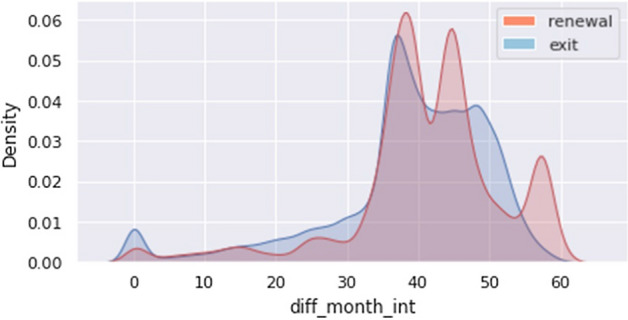
Results of comparative analysis by renewal (Y/N)—Rental usage days (usage_months)

First, the EDA results are explained for contract application (contract creation/termination time, rental model, and rental fee) and installation variables. Figure 6 shows the analysis result of the number of months of use, which is the difference between the contract end date and contract start date of rental customers, differing according to the renewal Y/N. Customers who leave (renewal_flag = N) peak at 36 months and customers who renew (renewal_flag = Y) tend to be prominent at 39, 45, and 57 months. According to the knowledge of the promotion calendar of the staff, 36 and 43 months are the starting times of the re-rental promotion, and the time of 3 months before the expiration (60 months) is also the time when the re-rental is mainly performed. However, it was confirmed that long-term loyal customers were maintained for more than 57 months.

**Fig. 7:**
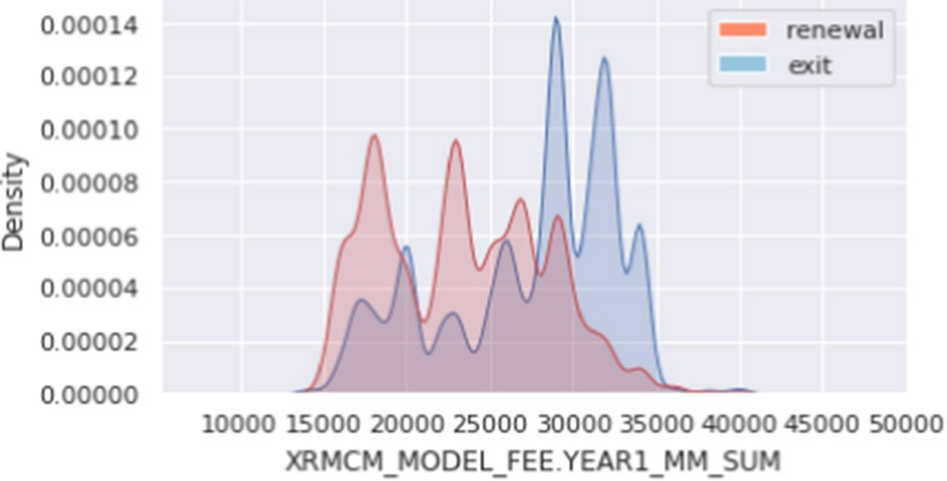
Results of comparative analysis by renewal (Y/N)—1st year monthly fee

Figure 7 shows a comparison of the first-year monthly fees between the Y/N groups. The churned customers are relatively more distributed in the section where the 1st year monthly fee is higher than that of the re-contracted customers.

**Fig. 8:**
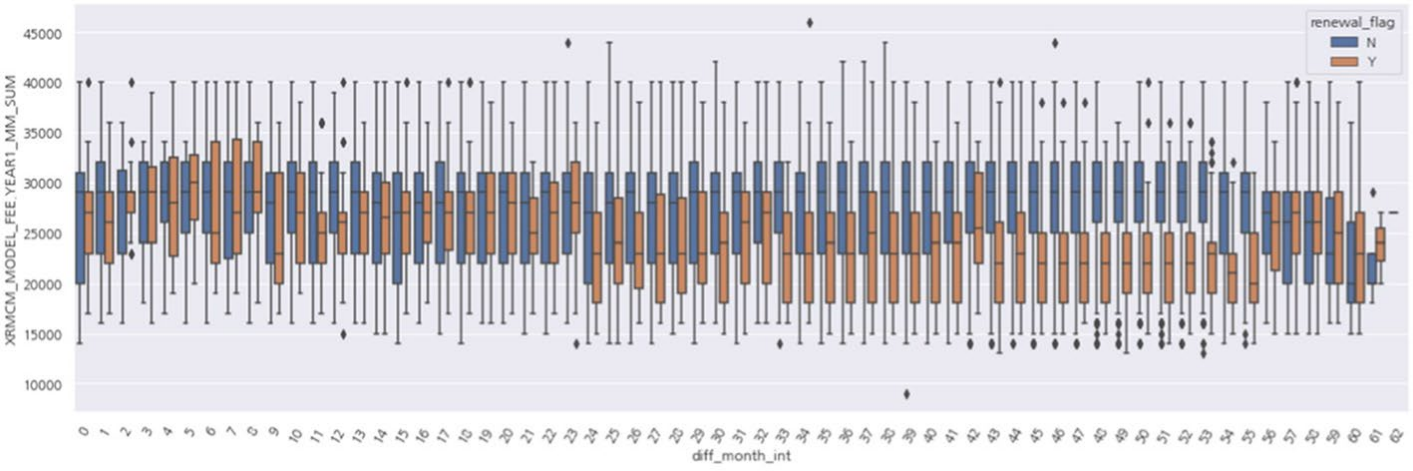
Results of comparative analysis by renewal (Y/N)—Monthly fee for the first year according to the number of months of use

Figure 8 shows the results of the comparative analysis between Y/N groups according to the number of months the service was used with the first-year monthly fee. As the number of months of use increases (up to 56 months), the monthly fee for the first year of the re-contracted customers decreases, but the monthly fee for the first year of the churned customers hardly changes. In addition, at 57 months, 3 months before the expiration date, there is a tendency that there is little difference in the rates between re-contracted and churned customers. In short, it can be estimated that the customers who churn the contract tend to have relatively high rental fees compared to the customers who renew.

**Fig. 9:**
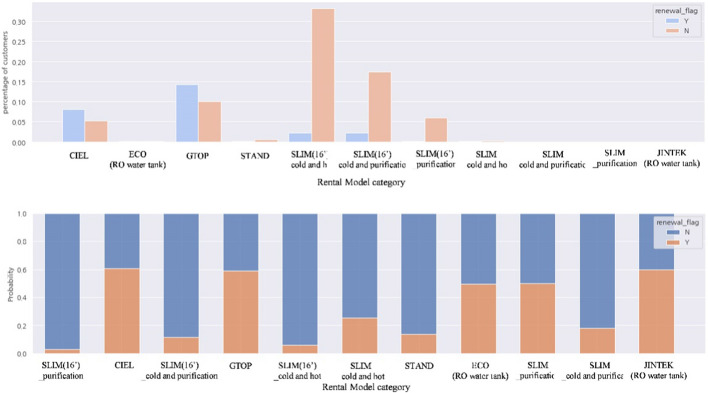
Results of comparative analysis by renewal (Y/N)—rental model category

Figure 9 shows the results of the comparative analysis of the Y/N groups according to the product model category of rental devices. The main models with a high percentage of churned customers were slim (2016 product model), cold and hot, slim (2016 product model)-cold, and slim (2016 product model)-purification. Product models with a high percentage of renewed customers were the GTOP and CIEL models. In the case of slim (2016 product model) series, it was confirmed by the staff that it was a model that caused contract cancellation a lot due to mold issues in introducing a cooling and heating function. In addition, the data analysis results showed that the GTOP and CIEL models were product models in which a renewal promotion policy was applied during the lease period. From these results, it was confirmed that the product model category of rental devices is one of the factors influencing customer churn.

**Fig. 10:**
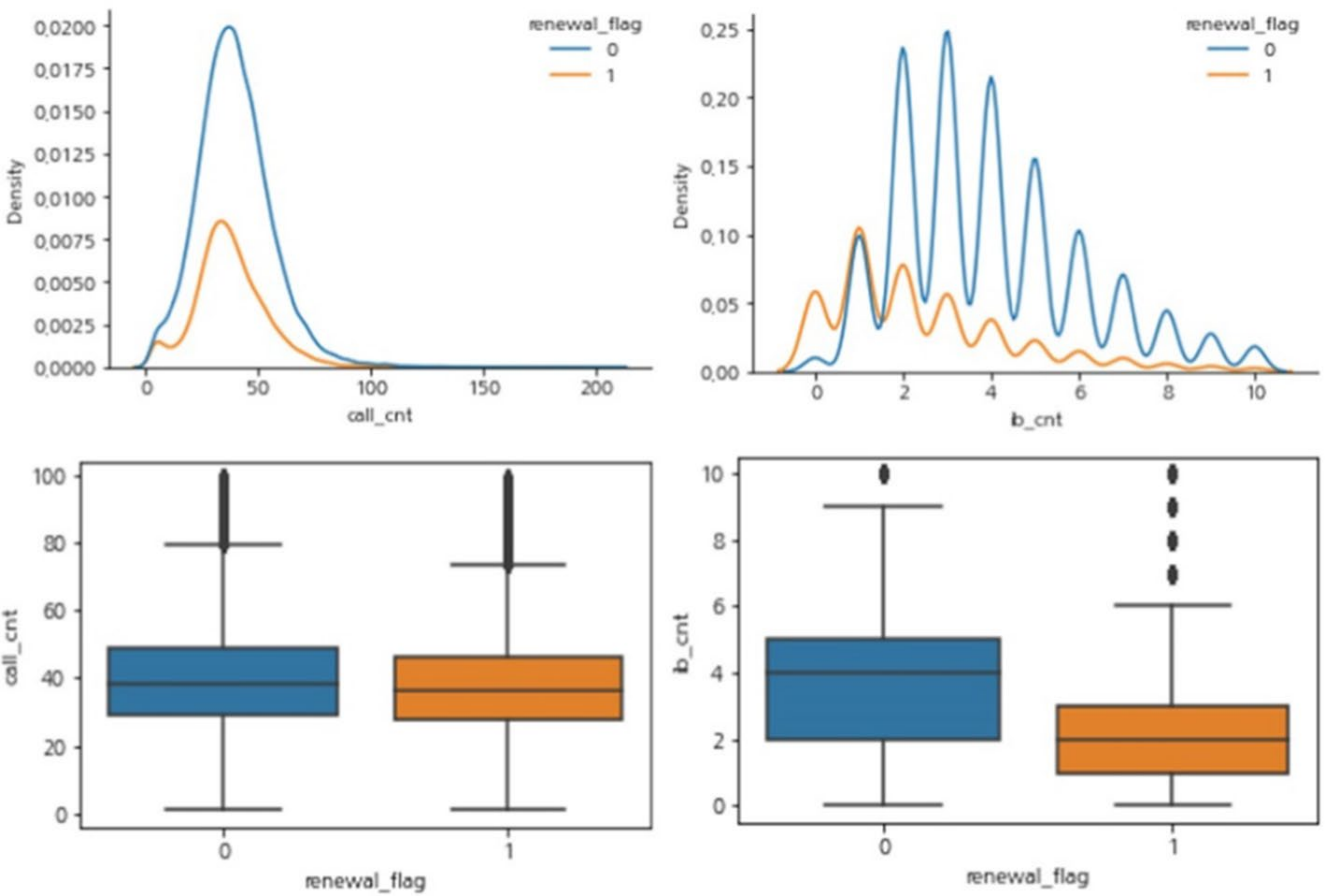
Results of comparative analysis by renewal (Y/N)—call count and inbound call count

Next, the EDA results for the call history (contact history) of customers are described. As the first feature of contact history, it was confirmed that discriminant power of the variable on the number of calls made per customer had a significant impact on the Y/N groups. Figure 10 shows the results of the comparative analysis by renewal (Y/N); call_cnt is the variable that records the total number of calls per customer, and ib_cnt is the variable that records the total number of inbound calls (calls from customers) per customer. Customers with a large number of call_cnt are less likely to renew their contracts, and filtering only inbound calls has a greater effect. In predictive modeling, it was confirmed that the F1-score slightly increased when inbound calls were added as a feature instead of the number of calls.

**Fig. 11:**
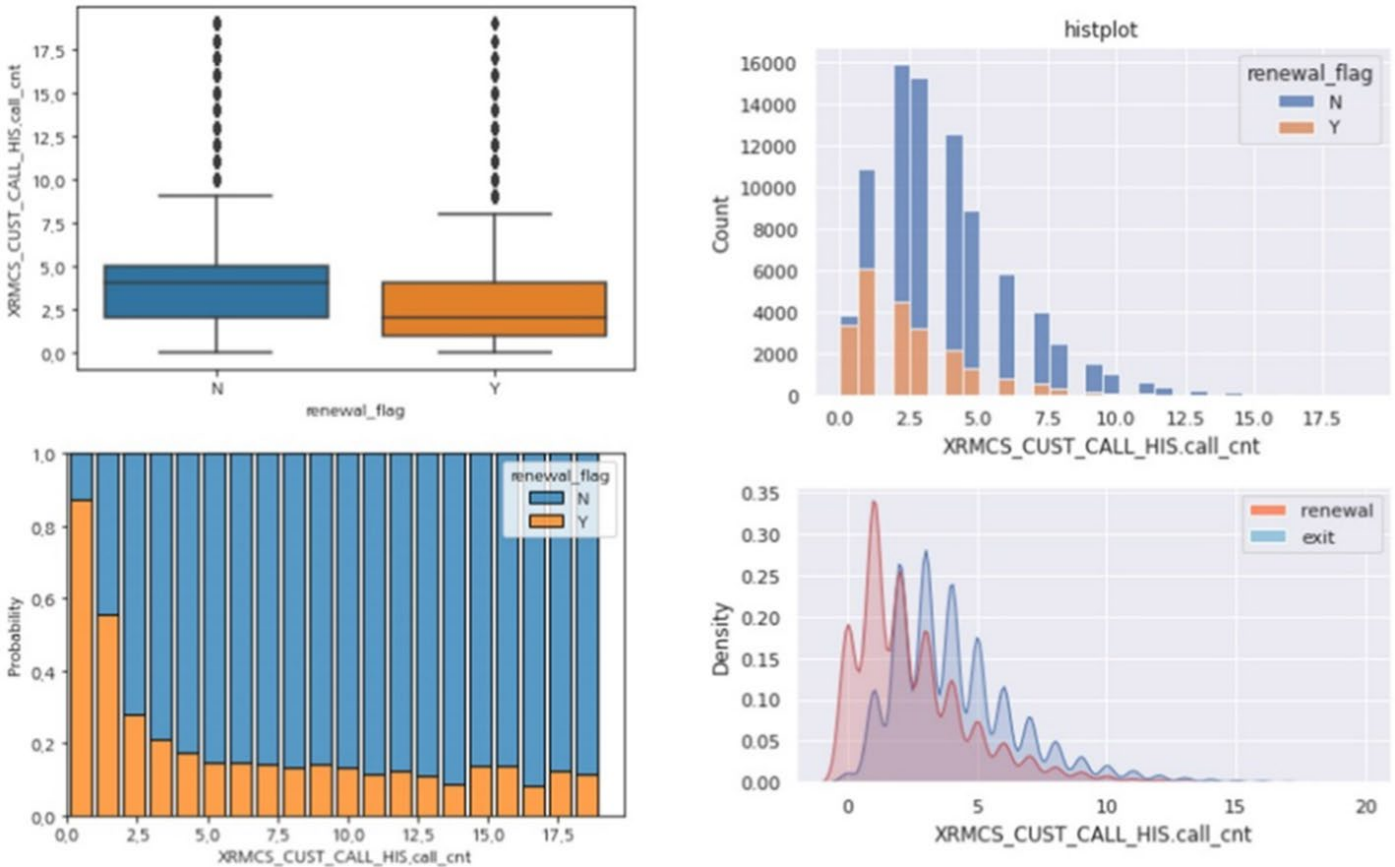
Results of comparative analysis by renewal (Y/N)—Inbound call count

Figure 11 shows the results of the variable that filtered only the inbound call count. It can be seen that the number of I/B call counts of churned customers is higher than that of re-contracted customers, and the recontract rate drops sharply by 2.5 times. In addition, 87% of customers who never made a phone call renewed their contracts, and 55% of customers who made just one call renewed their contracts. This can be interpreted as the effect that occurs when only the inbound call count is filtered, so contact history irrelevant to customer behavior, such as history or SMS, was removed.

Next, as the second feature of the contact history, a variable was created for the number of occurrences detailing call types for each customer. The call history-based attributes consist of the following four levels:

- 1st level (Call_History_GB): “I/B,” “O/B,” “History/SMS,” “ARS”
- 2nd level (Call_History_Type): cancellation related, product related, installation related, maintenance service, sales related, contract related, VOC management, customer promise, customer management, rental fee collection related, and direct water pipe replacement.
- 3rd level (Call_History_Group): churn defense (commitment discount), press/quality, service inquiry, recall related, churn defense (in-house resale), VOC, etc.
- 4th level (Call_History_Detail): relocation cost (pump cost) dissatisfaction/negligence, press/quality inquiry, moving, service inquiry, collection schedule inquiry, others, etc.

The type of call history can be distinguished by the combination of each of the above levels. For example, (history/SMS, contract related, care solution system/policy, system/policy inquiry) is one possible combination. The total number of combinations up to level 4 is 965, and the number of combinations up to level 3 is 136. Because it was confirmed that the combinations classifying up to Level 3 do not lose as much information as the combinations classifying up to Level 4, only combinations up to Level 3 were used to create a call type feature variable. In addition, it was confirmed that the data having a call type of churn defense (manager)’ or “cancellation reception” with a value of level 3 correlated very strongly with the target variable (churn or not). Considering that it is desirable to have a prediction model that detects a signal before the occurrence of cancellation as much as possible, these two values were removed to narrow the total number of combinations in level 3 to 130. Among them, only those features judged to show a significant difference between the Y/N groups were finally selected. Table 3 shows the finally selected call type features.

**Table 3:** Descriptions of the four call type features finally selected

Feature name	Descriptions
Call type 004	(“ARS,” “Maintenance Service,” “Others”)
Call type 008	(“I/B,” “contract related,” “contract information”)
Call type 020	(“I/B,” “fee collection related,” “fee collection”)
Call type 048	(“I/B,” “contract termination (cancellation) related,” “consultation on termination”)

Table 4 shows the results of the comparison analysis between the Y/N groups of the four call type features; call_type_004 features were created by the combination of (ARS, maintenance service, others); call_typ008 feature were created by the combination of (I/B, contract related, contract information); call_type_020 features were created by the combination of (I/B, fee collection related, fee collection); call_type_048 features were created by the combination of (I/B, contract termination (cancellation) related, consultation on termination). The call_type_004 feature showed that the renewal rate was lower for customers who made many inquiries about the maintenance service through the ARS. The call type 008 feature showed that the renewal rate of customers who frequently called to obtain contract information was low. The call_type_020 feature showed that the renewal rate was lower for customers who frequently called to inquire about billing. The call_type_048 feature indicated that the renewal rate was lower for customers who frequently called cancellation advice.

**Table 4:** Results of comparative analysis by renewal (Y/N) – call type features

Count	Renewa flag	Call Type 004 (%)	Call Type 008 (%)	Call Type 020 (%)	Call Type 048 (%)
0	0	0.66	0.66	0.72	0.69
	1	0.34	0.34	0.28	0.31
1	0	0.93	0.80	0.73	0.82
	1	0.07	0.20	0.27	0.18
2	0	0.95	0.84	0.78	0.85
	1	0.05	0.16	0.22	0.15
3	0	0.96	0.86	0.79	0.88
	1	0.04	0.14	0.21	0.12
4	0	0.97	0.87	0.77	0.85
	1	0.03	0.13	0.23	0.15
5	0	0.96	0.89	0.83	0.92
	1	0.04	0.11	0.17	0.08
6	0	0.99	0.88	0.85	1.00
	1	0.01	0.12	0.15	0.00

In addition to the four selected variables as a result of the analysis of significant differences between Y/N groups according to call type features, several other variables with interesting results were found. Table 5 shows the result of comparative analysis between Y/N groups of the call_type_096 feature created by the combination of (history/SMS, contract-related, care solution system/policy). Regrading care solution system/policy’, 13% of those who were informed once and 69% of those who were informed twice renewed their contract. This result is worth examining to determine whether the churn rate can be lowered if the company first informs customers of systems and policies at an appropriate time before customers make inquiries through inbound calls.

**Table 5:** Results of comparative analysis by renewal (Y/N)—call type 096

Count	Renewal flag	Proportion (%)
0	0	0.71
	1	0.29
1	0	0.87
	1	0.13
2	0	0.31
	1	0.69
3	0	0.58
	1	0.42
4	0	0.33
	1	0.67

Finally, the EDA results related to rental commitment, discount, payment, visit history, and repair history are described. The following is a description of the newly discovered features.

- Commitment-related features: commitment number, commitment amount, commitment start date, end date, etc.
- Discount-related features: type of discount, reason for the discount, time of discount, amount of discount applied, etc.
- Payment-related features: payment method, payment method, charge path, etc.
- Features related to visiting history: regular/irregular number of visits, number of times visit date and time changed, weekend/weekday visits, manager’s age, etc.
- Features related to product repair history: number of repairs, number of occurrences by repair type, etc.

### Feature Refinement

When a feature has a high correlation coefficient with another feature, a change in one feature causes a commensurate change in the highly correlated feature. Correlations between features can lead to multicollinearity and affect the performance of the model. In addition, it is recommended that important features showing a high correlation with the target class label be selected and other features be excluded. We conducted statistical data exploration with Open-source Python library. Specifically, we use Numpy and SciPy for computing correlation coefficients of variables and statistical models for computing variance inflation factors.

Therefore, for the classification model performance, the feature set was classified into 15 groups, as shown in Table 6, according to the meaning of the features to reduce redundancy that may exist among approximately 80 previously selected features and to select only the essential features. Finally, 53 features were selected by checking the multicollinearity between features within each group as well as between different groups, and comparing the correlation with the target class label.

**Table 6:** List of 80 selected feature set and finally selected 53 features

Feature group	Feature list	Selected	Format	Feature descriptions
1. Rental fee	Monthly discount amount	o	Numeric	Monthly rate discount
	Sale price	o	Numeric	Sale price
	Sale charge	o	Numeric	Sales fee
	Total rental fee	o	Numeric	Total rental amount
	1st Year Rental Fee, 2nd Year Rental Fee, 3rd Year Rental Fee, 4th Year Rental Fee, 5th Year Rental Fee		Numeric	Rental fee of 1st year (to 5th year)
2. Contact history (call count, call type history, etc.)	Call Count	o	Numeric	Number of inbound calls in DATE_DIFF(CURRENT_DATE(),CONTACT_DATE, Year) < 1))
	Call Type 004	o	Numeric	Number of calls of type (GB, TYPE, GROUP) = (“ARS,” “Maintenance Service,” “Others”) in DATE_DIFF(CURRENT_DATE(),CONTACT_DATE, Year) < 1))
	Call Type 008	o	Numeric	Number of calls of type (GB, TYPE, GROUP) = (“I/B,” “Contract related,” “Contract information”) in DATE_DIFF(CURRENT_DATE(),CONTACT_DATE, Year) < 1))
	Call Type 020	o	Numeric	Number of calls of type (GB, TYPE, GROUP) = (“I/B,” “Fee collection related,” “Fee collection”) in DATE_DIFF(CURRENT_DATE(),CONTACT_DATE, Year) < 1))
	Call Type 048	o	Numeric	Number of calls of type (GB, TYPE, GROUP) = (“I/B,” “contract termination Related,” “consultation on termination”) in DATE_DIFF(CURRENT_DATE(),CONTACT_DATE, Year) < 1))
3. Discount (applied amounts, reason category, type, etc.)	Discount application Amount_min	o	Numeric	Minimum value of discount application amount
	Discount application Amount_max	o	Numeric	Maximum value of discount application amount
	Discount application Amount_avg		Numeric	Mean value of discount application amount
	Discount reason code(40) Count		Numeric	Number of times “Reason for discount” is code “promotion”
	Discount reason code(77) Count		Numeric	Number of times “Reason for discount” is code “commitment discount”
	Discount reason code(90) Count		Numeric	Number of times “Reason for discount” is code “settlement of churn receipt balance”
	Discount reason code(10) count		Numeric	Number of times “Reason for discount” is code “customer dissatisfaction”
	Discount reason code(88) count		Numeric	Number of times “Reason for discount” is code “churn defense discount”
	Discount reason code_40	o	Yes/No	Whether or not the code value “promotion” of “Reason for discount” exists
	Discount reason code_77		Yes/No	Whether or not the code value “commitment discount” of “Reason for discount” exists
	Discount reason code_90	o	Yes/No	Whether or not the code value “settlement of churn receipt balance” of “Reason for discount” exists
	Discount reason code_10	o	Yes/No	Whether or not the code value “customer dissatisfaction” of “Reason for discount” exists
	Discount reason code_88	o	Yes/No	Whether or not the code value “churn defense discount” of “Reason for discount” exists
	Discount type ST count		Numeric	Number of times “discount type” is code “ST (normal discount)”
	Discount type SP count		Numeric	Number of times “discount type” is code “SP (special discount)”
	Discount TYPE SC Count		Numeric	Number of times “discount type” is code “SC (commitment discount)”
	Discount Type ST	o	Yes/No	Whether or not the code value “ST (normal discount)” of “Discount Type” exists
	Discount Type SP	o	Yes/No	Whether or not the code value “SP (special discount)” of “Discount Type” exists
	Discount Type SC	o	Yes/No	Whether or not the code value “SC (commitment discount)” of “Discount Type” exists
	Discount date diff from start	o	Numeric	The number of days between the current date and the maximum discount start date
4. Commitment (sequence, amount, date, etc.)	Commitment max amounts		Numeric	The maximum value of commitment amounts
	Commitment max sequences	o	Numeric	The maximum value of commitment sequences
	Commitment date diff from creation to start	o	Numeric	The number of days between the minimum commitment start date and the minimum creation date
	Commitment date diff from creation		Numeric	The number of days between the current date and the minimum creation date
	Commitment date diff from start		Numeric	The number of days between the current date and the minimum commitment start date
5. Usage days	Usage days	o	Numeric	The number of days between the current date and contract creation date
6. Visit history (scheduling style.)	Non period visit	o	Numeric	Number of irregular visits
	Period visit	o	Numeric	Number of regular visits
	Visit change		Numeric	Number of rescheduled visits
	Not visit	o	Numeric	Number of cancelled visits
	Weekend visit	o	Numeric	Number of weekend visits
7. Manage age (Rental door-to-door service manager age)	Manager Age_min		Numeric	Minimum age of manager the customer encountered
	Manager Age_max		Numeric	Maximum age of manager the customer encountered
	Manager Age_avg	o	Numeric	Average age of manager the customer encountered
	Manager Age_range		Numeric	Range in age of manager the customer encountered
	Manager Age_std	o	Numeric	Standard deviation in age of manager the customer encountered
	Last Manager Age	o	Numeric	The age of the last manager the customer encountered
8. Repair history	Normal repaiR	o	Numeric	Number of “general type” repairs received
	Shibang repair	o	Numeric	Number of “shibang type” repairs received
	Heavy repair	o	Numeric	Number of “heavy type” repairs received
9. Rental model category	Rental model category	o	Categoric	Categories such as “GUBUN1_ SLIM(16’)_cold and hot,” “GUBUN1_GTOP,” “GUBUN1_SLIM(16’)_cold and purification,” “GUBUN1_CIEL,” “GUBUN1_SLIM(16’)_purification,” “GUBUN1_ECO(RO water tank),” “GUBUN1_STAND,” “GUBUN1_JINTEK(RO water tank)”
10. Sales type	Sales type		Categoric	Sales channels such as home appliance store, personal sales, electronic land, commercial specialty store, hi-mart, hi-care, home shopping, etc
11. Rental model color	Rental Model Color	o	Categoric	Colors such as “PROGRAM.COLOR_luxury white,” “PROGRAM.COLOR_white,” “PROGRAM.COLOR_silver,” “PROGRAM.COLOR_black,” “PROGRAM.COLOR_red,” “PROGRAM.COLOR_luxury black,” “PROGRAM.COLOR_shine,” “PROGRAM.COLOR_gray”
12. Rental model function	Rental model function	o	Categoric	Functions such as “PROGRAM.FUNCTION_hot/cold/purification,” “PROGRAM.FUNCTION_purification,” “PROGRAM.FUNCTION_cold/purification,” “PROGRAM.FUNCTION_hot/purification,” “PROGRAM.FUNCTION_hot/cold/week cooling”
13. Delivery goods type	Order type (e.g. DELIVERY_GOODS_TYPE_TypeA)	o	Categoric (15 values)	Delivery type such as re-rental after 3 years of use (additional discount), home appliance package, re-rental for 5 years (before expiration/additional discount), plus (50,000 won /prepayment exemption), plus (100,000/5000 won discount), water tank type replacement (re-rent for less than 3 years), etc (Select the values (SELECTED_VALUES) in which the ratio of the value count of the DELIVERY_GOODS_TYPE variable is 1% or more and create a boolean variable (DELIVERY_GOODS_TYPE_TypeA) for each cont_line_seq (e.g. “TypeA” in SELECTED_VALUES))
14. Transfer type	Transfer Type Transfer Change Route		Categoric categoric	Payment methods such as bank transfer, cash payment, deposit without bankbook, credit card transfer, etc
15. Receiver address	Address_1st offset Address_2nd offset		Categoric categoric	Address of first offset of full address Address of second offset of full address

As mentioned earlier, features with large values of correlation coefficients of pairs in Xs and variance inflation factors of Xs were removed. At the same time, features with large values of correlation coefficients with the target class label were selected as the significant features. Table 7 shows the results of the correlation coefficient between the finally selected features and the target class label. In Evaluation results Sect, the results of the analysis of the impact of feature refinement on classification performance are explained.

**Table 7:** The correlation coefficient between the finally selected features and the target class label

Feature list	Coefficients
Rental Model Category Usage days Rental Model Color Sale Price 4th year rental fee 5th year rental fee 1st year rental fee 2nd year rental fee 3rd year rental fee Total rental fee Commitment date diff from creation Commitment date diff from start Call Count Monthly discount amount Call Type 004 Sale Charge Manager age_max Last Manager Age Manager age_mean Call Type 008 Discount reason code_40 Manager Age_min Manager Age_range Call Type 048 Not Visit Manager Age_std Sales Type Weekend Visit Non Period Visit Discount date diff from start Discount application amount_max Discount application amount_mean Discount type ST Period visit Visit Change Discount Type SP Discount Application Amount_min Rental model function Discount reason code_90 Call Type 020 Transfer change route Transfer Type Discount reason code_10 Discount reason code_88 Address_2nd offset Address_1st offset Commitment max sequences Normal repair Commitment date diff from creation to start Shibang repair Heavy repair Discount reason code_77 Discount type SC	0.5 0.493 0.452 0.335 0.334 0.334 0.331 0.331 0.331 0.325 0.277 0.276 0.275 0.2 0.182 0.169 0.142 0.136 0.135 0.133 0.133 0.125 0.12 0.117 0.116 0.116 0.115 0.109 0.107 0.098 0.096 0.095 0.094 0.09 0.089 0.084 0.078 0.052 0.048 0.046 0.038 0.038 0.035 0.026 0.017 0.016 0.015 0.007 0.004 0.002 0.002 0.001 0.001

## Evaluation results

In describing the performance of the classification models, Label Y refers to a customer who is about to leave without using the service for some reason, and label N refers to a customer who is satisfied with the service and wants to continue using it.

### Evaluate the performance of classification models

**Fig. 12:**
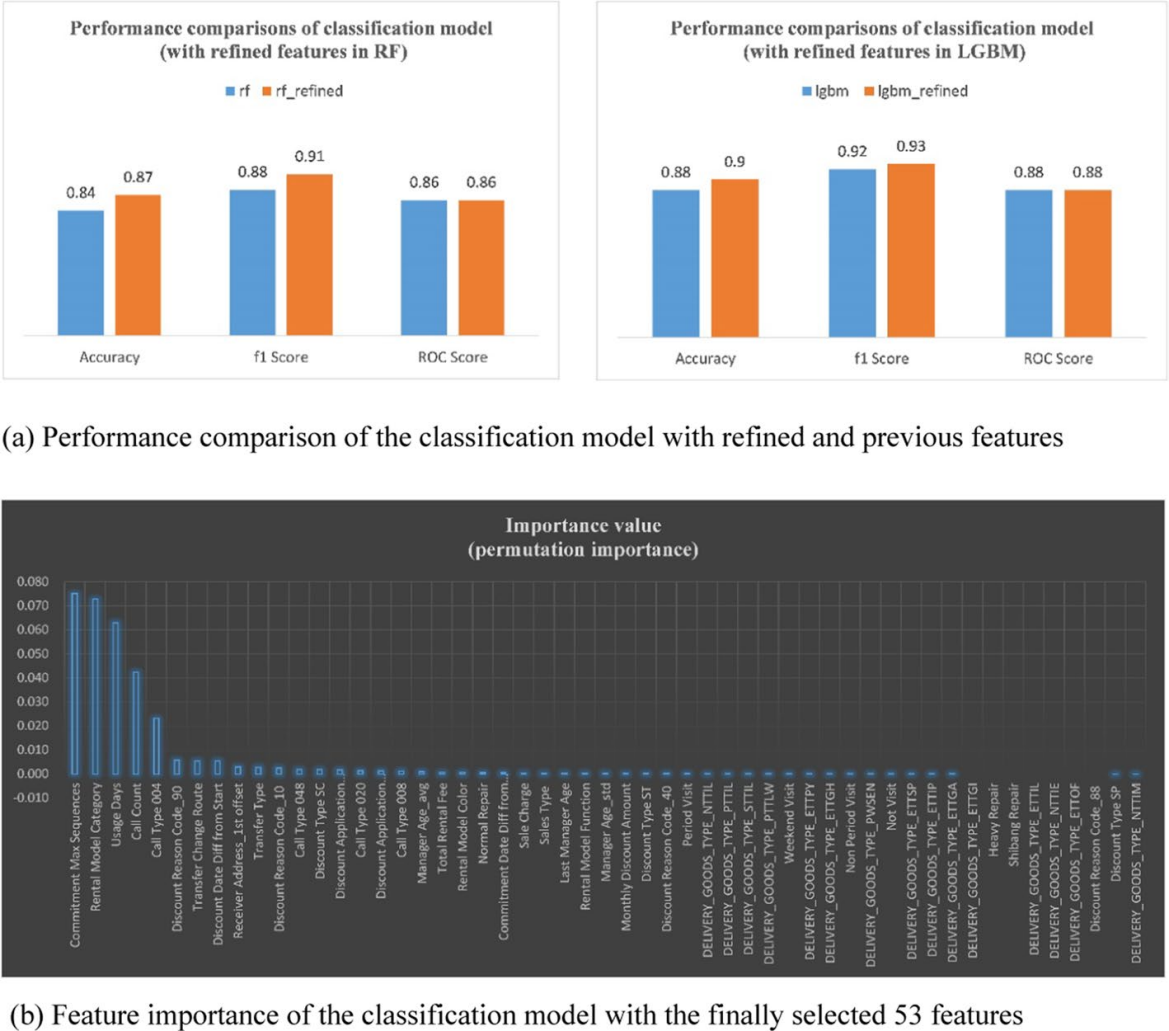
Performance and feature importance of the classification model with the finally selected 53 features. **a** Performance comparison of the classification model with refined and previous features. **b** Feature importance of the classification model with the finally selected 53 features

First, the impact of the feature refinements mentioned in Feature Refinement Sect on classification performance was evaluated. Figure 12 compares the performance of the classification model between 53 refined and 80 regular features to help determine the performance improvement achieved using the refined features of the classification model. Although there was no difference in performance in the AUC score, there was a difference in model performance accuracy with 90% and F1 score with 93% using the 53 significant features selected based on correlations between features compared to using the information in the 80 regular features (accuracy: 88% and F1: 92%). Figure 12(b) shows the importance of scores based on the permutation of the finally selected 53 features. As illustrated in Fig. 12(b), the top five features that affect customer churn are: commitment max sequences, rental model category, usage days, call count, and call type 004.

In the case of the maximum number of commitment sequences, similar to usage days, regarding the difference between the current date and the contract creation date, the longer the customer used the service, the higher the probability of contract renewal was. The probability of customer churn varied depending on the rental product model. The first factor related to the model was whether the model was included in the company's rental promotion. The second factor was whether there was a specific model that caused product defects. Customer’s contact history was also found to be an important factor. The greater the total number of inbound calls from customers in the past year, the higher was the risk of customer churn. Compared to outgoing calls, incoming calls are a means of expressing the customer’s wishes, especially dissatisfaction. In addition, customers who place more calls for ARS/maintenance services/other have a higher risk of churn. This means that the customer’s service satisfaction with ARS-type maintenance service-related inquiries is low and the need to improve service quality can be confirmed.

Thus, the final selected 53 features were used in the machine learning algorithms to identify the customers at risk of churn. Using data from 84,000 customers who rented water purifiers in 2016, two different algorithms were tested based on an ensemble in combination with three different feature sets. The feature set for comparative analysis was composed of the following three groups: all feature sets (Algo1), baseline feature sets, and sets (Algo2) excluding baseline features from all feature sets. To select the criteria for the baseline algorithm, the information previously used to prevent customer churn was inquired of rental care customer management staff. The number of months of use was almost the only criterion mentioned by rental care service managers in judging the possibility of customer cancellation, and the number of cancellations started to rise after 36 months of the mandatory contract period. Therefore, a model predicting using only the number of days of use as a feature was selected as the baseline model.

Thus, the performance of our prediction model was compared with the models with “usage days” related features. A baseline model with “usage days” related features indicates the increase in termination rate as the customer’s mandatory contract period approaches. Different random forest (RF) and light gradient boosting machine (LGBM) models such as the full-feature-based model which learns all the selected features and the sub-feature-based model which learns the remaining features except for "usage days" were also considered.

**Fig. 13:**
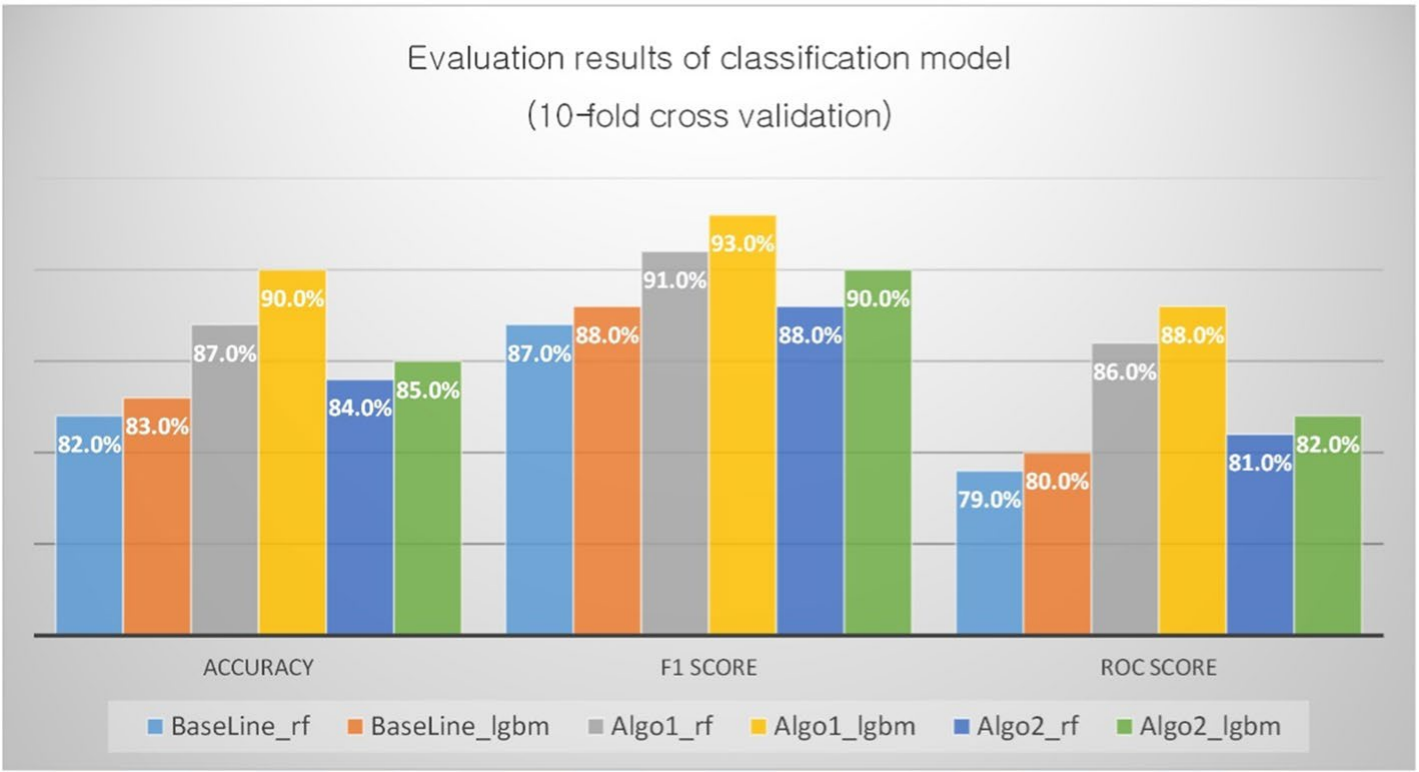
Evaluation results of classification models—3 algorithms in RF vs. LGBM

The bagging approach decreases the variance in the prediction by generating additional data for training from the dataset using combinations with repetitions to produce multiple sets of the original data. The boosting approach iteratively produces improved models that more heavily weigh the impact of classification errors made by the previous iteration. In this technique, consecutive trees (random samples) are fit, and at every step, the goal is to improve the accuracy of the prior tree. The baseline and proposed methods were compared based on these two methods. The baseline model is the classification model with the three "usage days" related features which consist of commitment max sequences, commitment date diff from creation to start, and usage days. The Algo1 and Algo2 models each have 53 full-set features and 50 features, excluding "usage days” related variables. The classification model was evaluated with cross-validation (tenfold) and the results of each classifier-feature selection combination are shown in Fig. 13.

The baseline feature sets with RF and LGBM performed poorly, with accuracies of 82% and 83%, respectively. The Algo1 feature set with both RF (87%) and LGBM (90%) outperformed the Algo2 feature set with RF (84%) and LGBM (85%). As an indicator for performance evaluation, the F-measure value, which is a more meaningful accuracy measure considering false negatives and false positives, was calculated. In the case of false negative (FN) that did not detect customers who were thinking of leaving, serious damage is implied to the company business because of decreasing customer residual rate. Conversely, in the case of false positives (FP), which incorrectly predict model customers who do not intend to leave, as leaving the company, leads to spending funds on unnecessary promotions.

In addition, the AUC value, which is a measure of whether stable predictions can be made was calculated to distinguish labels while being less sensitive to the decision boundaries. An intuitive interpretation of the resulting AUC value is that it provides an estimate of the probability that a randomly chosen instance of class “churn” is correctly ranked higher by the classifier than a randomly selected instance of class “non-churn.”

In the case of F-measure, the baseline feature set with RF and LGBM performed poorly with F-measures of 87% and 88%, respectively. The F-measures for Algo1 and Algo2 features with both RF and LGBM were 91% for RF and 93% for LGBM for Algo1, 88% for RF, and 90% for LGBM for Algo2. In terms of AUC, the baseline feature set with RF and LGBM also performed poorly with AUC scores of 79% and 80%, respectively. The AUC score for Algo1 and Algo2 feature sets with RF and LGBM were 86% for RF, 88% for LGBM for Algo1, 81% for RF, and 82% for LGBM for Algo2. Algo1 was the best, and Algo2 was better than the baseline in both LGBM and RF.

**Fig. 14:**
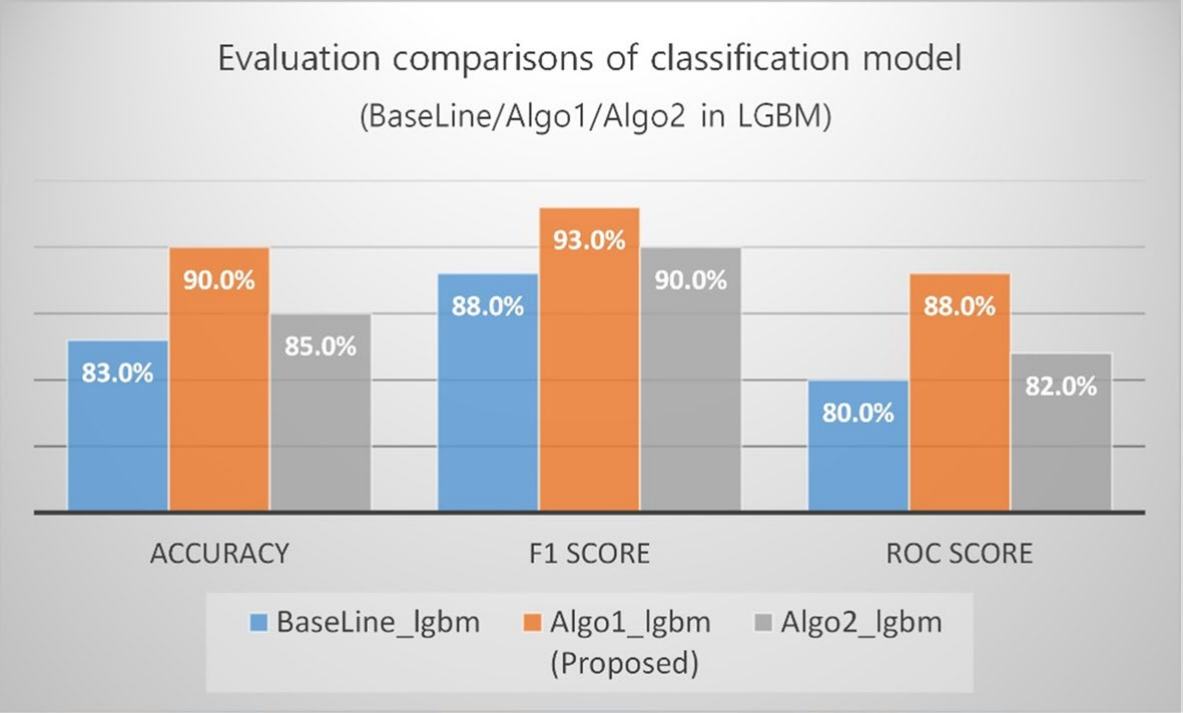
Evaluation comparisons of classification models—Baseline/Algo1/Algo2 in LGBM

The comparison results of the model performance in terms of the bagging and boosting methodologies are as follows. In all models (baseline, Algo1, and Algo2), LGBM performed better than RF (in terms of accuracy, F1, and AUC score). As shown in Fig. 14, for RF and LGBM of Algo1, LGBM significantly outperformed RF in terms of accuracy (t-test statistic = 24.77, p < 0.001).

The difference in the mean of the two classifiers was almost zero in the case of the F-measure and AUC scores, so these two classifiers were not significantly different. The choice of RF or LGBM depends on the selection of the machine learning algorithm to adopt using the selected features (Algo1), so the statistical significance of the performance difference between F1 and the AUC score is not very important.

LGBM-based modeling showed better results than baseline for both Algo1 and Algo2, based on statistical significance. The details on the statistical significance are presented in Table 8.

The findings of the feature importance are as follows. Among the top three features of importance in ranking Algo2, the first is call count; the second is call_type_004; and the third is rental model category. That is, it can be confirmed that the dominant feature among the remaining features, except for the usage day-related variable, is the variable related to the customer's contact history.

### Evaluate the inference performance of predictive models

To measure the field performance of the predictive model, currently, a pilot test is being conducted to infer the churn probability of the test target customers. To verify the effectiveness of measuring the performance, the predictive model in this study was applied to the actual service of the company planned for the future. Concept drift was considered when selecting the test target data to measure the performance of the predictive model. “Concept drift” is a term used in the field of machine learning and refers to a phenomenon in which the statistical characteristics of a modeling target change over time. (https://en.wikipedia.org/wiki/Concept_drift).

First, considering concept drift, the most recent time point of the test data that should be used to measure the performance of the predictive model was selected. The selection criterion was the number of months of use in which most terminations occurred. Accordingly, customers contracted from January to August 2018 (36 to 43 months based on the number of months of use) were selected. Based on 84,000 accounts of learning data, the average monthly churn rate was approximately 0.5% in the period prior to 36 months, where churn incurs a certain penalty in fee. In the periods after 43 months, the average churn rate was approximately 2.5%. These two sections tend to have a relatively low churn rate compared to the 36-month to 43-month section, which has an average churn rate of approximately 5%.

Next, the sensitivity to concept drift of the data to be modeled was checked. The difference between the test data and learning data was that the “yearly water pipe replacement service” was added to the service content. In addition, it was confirmed that the functions of the water purifier (cold water, hot water, water purification, etc.) and rental service content (visit frequency, water quality inspection, service content such as filter replacement, parts cleaning, etc.) were almost similar.

#### Monitoring contract status change—hit rate

**Fig. 15:**
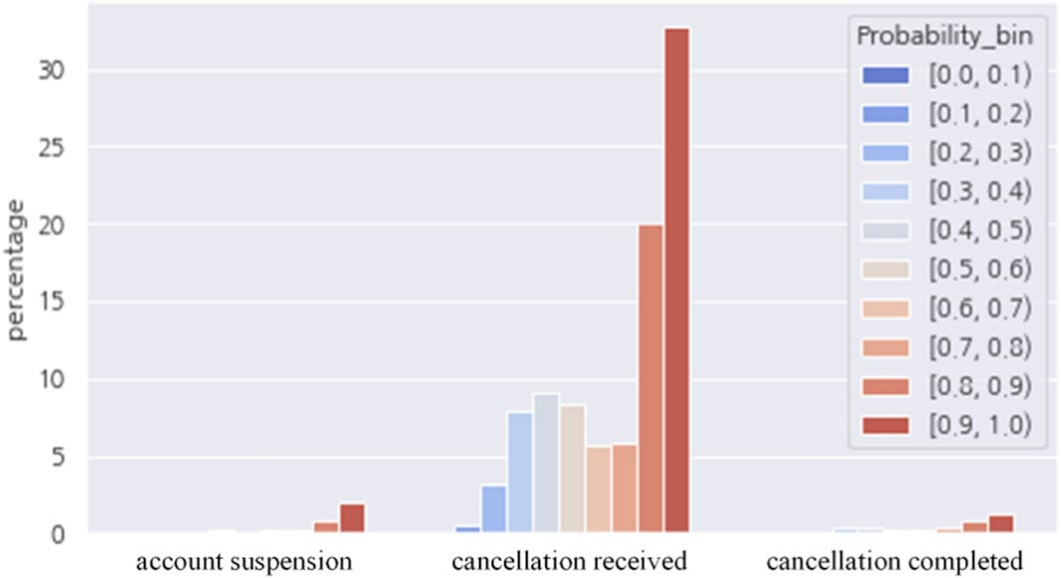
Proportion of number of customers by prediction probability according to contract status

Based on the probability predicted by the classification model, the prediction accuracy of the number of customers was derived. The test subjects were 256,234 water purifier customers contracted from January to August 2018, and the monitoring period for counting was approximately four months, from August 7, 2021, to December 13, 2021. Whether the customer left or not was confirmed by checking the contract status changes of 11,972 customers from “normal” to “account suspension,” “cancellation received,” and “cancellation completed.” Because the contracts were not affected by the discount policy (re-rental for 36 months), change of contract status to "cancellation" means not cancellation for renewal of contract but one for churn from service. There were 9,456 customer accounts that matched the customers predicted by the classification model as “churn”. confirming a hit rate of about 79%. Figure 15 shows the proportion of the number of customers by the prediction probability bin according to the contract status.

Based on the probability value of the predictive model, the actual number of customers who were tested (about 250,000) showed a distribution of about 55% in the section above 0.8 with the following two performance indicators: “churn detection power” and “churn LIFT.”

- Churn detection power: Top 10% customer hit rate
- Churn LIFT: The measure of how many times the churn rate in the top 10% is higher than the average churn rate (number of churned customers/number of accumulated customers).

In the case of a telecommunication company, targeting customers who are most likely to churn in the month of campaign activity, customers to churn in the M + 2 months were predicted. Although refinement is necessary to fit the rental care service domain, as it is an indicator of the churn rate of the telecommunication company, the predictive model performance of the pilot experiment was benchmarked in designing the performance index. The churn detection power of our test target customer was about 39%, and the churn LIFT could be explained as about 1.2 (average churn rate 3.8, churn rate 4.1 in the top 10%). However, because the prediction accuracy varies depending on the number of prediction targets and the prediction cycle in the actual future operating system, it is necessary to consider these differences in the pilot currently running. It is also necessary to provide guidelines on the thresholds of the two indicators for sufficiently good performance.

#### Performance comparison with random group

As another method of predicting model performance, both a group of N high-risk individuals based on predictive models and a group of N individuals based on random sampling were configured from among the total test target customers (approximately 250,000). The matching ratio was then calculated by comparing with customer accounts that canceled the contract in each group. First, an experimental group A was formed by extracting N customer accounts with high risk of churn based on a predictive model, and a control group B was formed by randomly extracting N accounts from 250,000 customer accounts. Next, the refined experimental group (A’) was constructed by excluding the accounts from group A overlapping with group B, and the refined control group (B’) was formed by excluding from group B the accounts overlapping with group A. Sampling was performed a sufficiently large number of times (500 times) to construct the control group, and the average matching ratio between the prediction model group (A’) and the random group (B’) with the actual cancellation accounts was aggregated. As a result, the average matching rate of the prediction model group (A’) among 45,383, excluding the 151917 duplicate accounts in both groups, was 18.17%. The average matching rate for random group (B’) was 16.19%. It was confirmed that the difference of 1.98% in the average matching rate between the two groups was statistically significant. (t-test statistic = 14.29, p = 0.0002), respectively.

### Interpretation of modeling results

#### Global model-agnostic methods: partial dependence Plot

The discussion above focuses on the performance of the classifiers, which is only one aspect of a good churn prediction model. The interpretability of the resulting classifiers is also a key property because the comprehensibility of the model is important. The partial dependence plot (short PDP or PD plot) shows the marginal effect of one or two features on the predicted outcome of a machine-learning model [33]. The partial dependence plot considers all instances and explains the global relationship between the feature variable and predicted outcome.

**Fig. 16:**
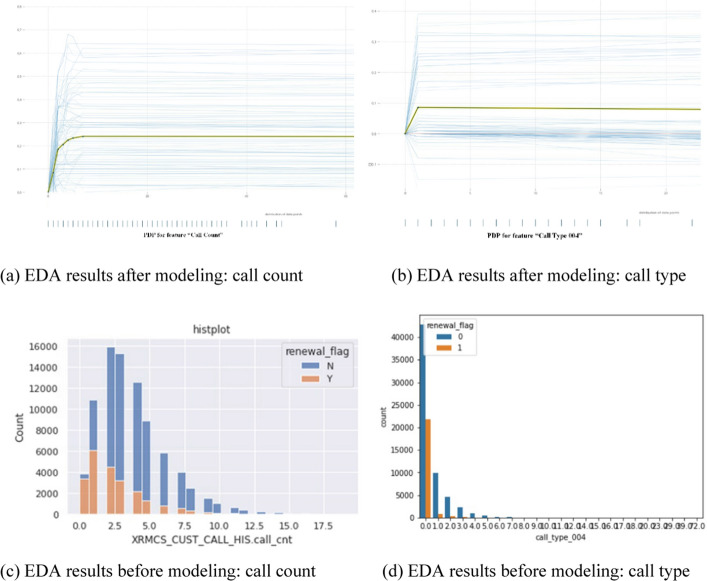
Analysis result of Call Count and Call Type 004 variable. **a** EDA results after modeling: call count. **b** EDA results after modeling: call type. **c** EDA results before modeling: call count. **d** EDA results before modeling: call type

Figure 16 shows the PDP of churn probability according to the number of call counts. It is observed that the effect on churn increases rapidly until approximately three calls and then gradually decreases, and there is no significant change after approximately 10 calls. That is, it is possible to check the range of the high influence of call count on each of the target variables Y/N. This shows the same trend as in the analysis results obtained from the EDA process before learning with the classification model. In other words, it is observed that the number of I/B calls from churned customers is higher than that of re-contracted customers, and the rate of renewal of contracts sharply decreases in the range of 1 ~ 2.5 calls. Fig. 16 also shows the PDP of the churn probability according to the call type 004 feature generated by the combination (ARS, maintenance service, others). In the case of this call-type variable, the influence of churn increases significantly within the first three calls and shows the same trend as the EDA result before modeling. In other words, the renewal rate is lower for customers who have inquiries about maintenance services through ARS.

**Table 8:** Statistical significances—Algo1 and Algo2 with Baseline

Approach (models)	Performance metric	Statistics	p-value
baseline and algo1	accuracy	− 36.95	< 0.001
	f-measure	− 35.51	< 0.001
	ROC score	− 37.38	< 0.001
baseline and algo2	accuracy	− 8.56	< 0.001
	f-measure	− 8.29	< 0.001
	ROC score	− 8.54	< 0.001

#### Post-Care activities using model-based EDA results

Table 9 shows an example of a test operation strategy for active churn defense that can be executed first using the dominant features derived from our churn prediction model. Promotional activities and follow-up management methods using the information on high risk churn include available features, such as the number of months of use and contact history, which rank among the top three key features of the predictive model mentioned in Evaluate the performance of classification models Sect. First, a CRM promotion activity is performed to provide bundled products to customers who have inbound calls more than a certain number of times. In addition, it is possible to check whether complaints or problems have been satisfactorily resolved for customers who have placed many ARS-based calls of a specific type, to take the necessary actions on those issues.

**Table 9:** Proposal to test operation of care activities for customer churn prevention

Type of care activity	Criteria for subject extraction	Care activity
CRM promotion that offers to sign up for combined products for customers with high inbound calls	Common Criteria: Customers who have been in use for more than 36 months and whose contract status is “normal” Specific Criteria: About 2,000 people with 3 or more inbound calls without a combined account	CRM promotion that proposes products to be purchased or replaced as rental combination products by linking target customers’ home appliance purchase histories After performing a care activity, the effectiveness of the activity is tested by monitoring whether it is combined or purchased
Follow-up care for customers with specific types of call history with high churn relevance	Common Criteria: Customers who have been in use for more than 36 months and whose contract status is “normal” Specific Criteria: About 200 people who have ARS/maintenance service contract/ until October 21, but no commitment discount history	Divided into two groups of 100 people each, group 1 performs O/B calls and group 2 sends text messages to ensure that any inconveniences or inquiries are successfully resolved during ARS calls After carrying out care activities, customer behavior patterns (contact history, contract status) are monitored to test the effectiveness of the activities

#### Local explanation: derivation of major churn factors for visualization of single prediction

**Fig. 17:**
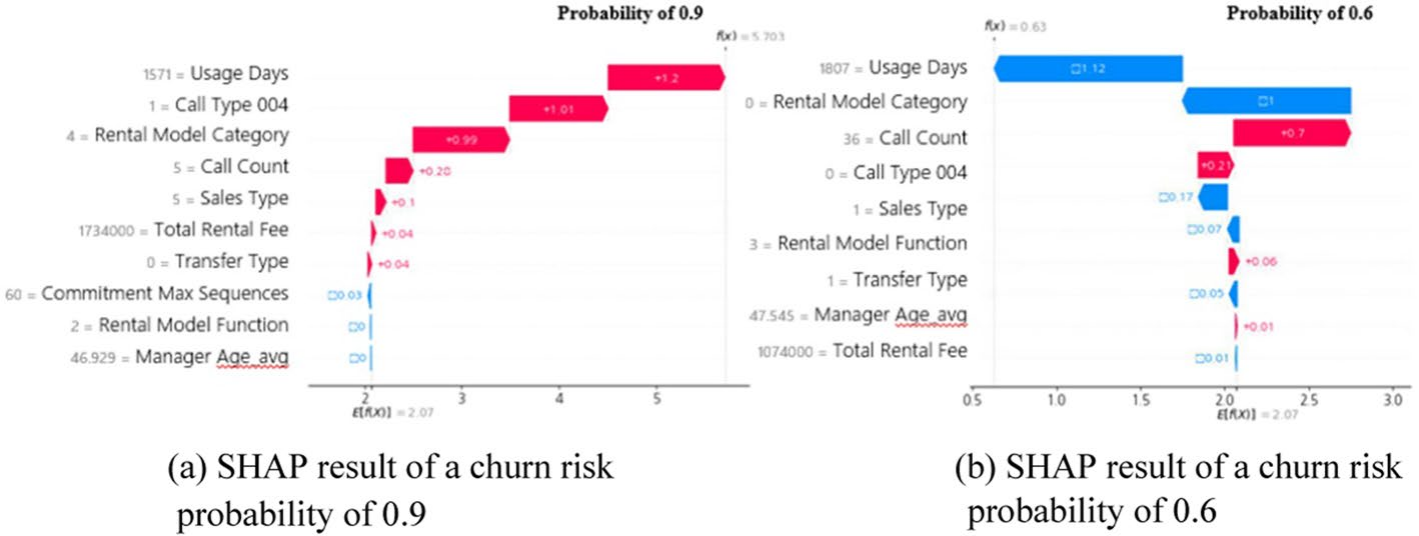
SHAP-based analysis results. **a** SHAP result of a churn risk probability of 0.9 (**b**) SHAP result of a churn risk probability of 0.6

The partial dependence plot described above only calculates the average influence of each variable on the results predicted by the model and has a limitation in that it cannot provide a method to explain the deviations among individual customers. Using SHAP, the predictive influence of variables in the model affecting each customer was identified. SHapley Additive exPlanations (SHAP) is a technique borrowed from game theory to determine a rational distribution method with Shapley value [32]. First, dominant feature items were derived using permutation importance based on the current prediction model and the feature items desired by a marketing person were additionally included. For the items configured in this way, the impact figure on the “churn probability” for each observation (customer) was calculated based on SHAP. Figure 17 shows the major SHAP-based churn factors of two customers, one with a churn risk probability of 0.9 and the other with a churn risk probability of 0.6.

The marketing organization to establish segmented target marketing strategies, utilizes the information on the possibility of churn and the cause predicted in advance for each customer. Thus, it is possible to carry out personalized churn defense activities for each customer.

## Conclusion and future works

A churn prediction study was conducted based on the customer behavior information of actual water purifier rental company, where customer churn occurs frequently because of the characteristics of customers who use the rental business. This study has academic significance in that the churn prediction model was validated by applying a machine learning algorithm to quantify churn risk information, and customer contract information was monitored during the operations. In particular, in the field, customers are prevented from leaving through group-level defensive activities, such as imposing a minimum number of months of use or through product groups that have not been differentiated for each customer. Through this study, based on a machine learning-based churn defense tool that effectively learns and predicts a customer's churn potential, it is possible to conduct churn prevention marketing in advance for customers with a high churn probability.

In terms of the performance indicators of predictive models in customer churn prediction, it was determined that there are factors to consider which are different from many other fields utilizing machine learning. In the case of false negatives (FN), which do not detect customers who are thinking of leaving, the prediction model could not predict churn. Thus, those customers would be left unattended without any defensive action against churn. This is a major obstacle to the company's goal of averting churn to increase the customer residual rate. In contrast, in the case of false positives (FP), the predictive model incorrectly detects that customers who do not intend to leave will leave. The customers receive some compensation through promotion because of incorrect predictions. However, promotions and advertisements incur costs, increasing expenditure costs as target size increases. Therefore, as the number of customers corresponding to FP increases, the expected profits from preventing churn decrease. The first goal is to increase the TP of the prediction model or reduce the FN to increase the residual rate by applying the churn prediction model. However, it is also important to reduce FP, which affects promotion costs for churn defense. In addition, from the perspective of data analysts, it is impossible to control the effectiveness of promotions that affect customer conversion rates. Evaluating the performance of the model from the perspective of detecting the signs of customer churn with a high expected value is meaningful future work in terms of expected profits.

A limitation of this study is that customer information from an external data source, that can improve prediction performance, was not used as a feature. In general, customer loyalty is formed through satisfaction with the service of the company but loyalty can also be lowered by attractive offers of better service from other companies. In the dataset used, there was no information on other external factors that stimulated customer churn. A prime example is whether a customer is tempted by a competitive deal from another rental service provider that offers a similar product at a lower rental cost. This study confirms the effectiveness of customer churn prediction modeling using customer data related to rental contracts, installation, operation, and maintenance. However, if customers' water purifier usage behavior data, such as daily usage frequency, usage time, and amount of water, are added as features to predictive modeling, the predictive power of customer churn can be further improved.

In addition, SHAP value, designed based on game theory, was applied to solve the unexplainable aspects with the existing machine learning model, reflecting on the domain knowledge of the person in charge of the business to derive the main reasons for customer churn. In other words, this study not only identified the major variables that affect churn, but also estimated the cause of churn for each customer to assist the marketing person in customer-tailored marketing.

It is expected that actual customer marketing activities such as qualitative care, CRM activities, and the application of differentiated costs for each customer are options for paid intervention to preclude customer churn. The verification results of the effectiveness, including expected profits, will be explored in future work.

## Data Availability

The dataset analyzed during the current study is not publicly available due to information security regulations prohibiting external disclosure of internal customer data within the company where I am currently employed.
